# Supplementary material for: Programmed Aptamer Screening, Characterization, and Rapid Detection for α-Conotoxin MI
Source: Toxins (Basel). 2022 Oct 14;14(10):706. doi: 10.3390/toxins14100706 (PMC9606946; doi:10.3390/toxins14100706)
Supplement: Supplementary file 1 [file toxins-14-00706-s001.zip › toxins-1947148-supplementary.pdf]

toxins

MDPI

Article

Programmed Aptamer Screening, Characterization, and Rapid Detection for α-Conotoxin MI

Han Guo, Bowen Deng, Luming Zhao, Yun Gao, Xiaojuan Zhang, Chengfang Yang, Bin Zou, Han Chen, Mingjuan Sun, Lianghua Wang and Binghua Jiao

Table S1. MB-SELEX conditions for aptamer screening.

Round	Amount of ssDNA library (pmol)	Amount of positive MBs (µl)	Amount of negative MBs (µl)	Incubation of positive rounds (min)	Incubation of counter rounds (min)	Wash after incubation (min)
1	1300	200	\	120	0	15
2	100	200	\	120	0	15
3	100	150	\	90	0	30
4	100	150	\	90	0	30
5	100	100	\	60	0	45
6	100	100	\	60	0	45
7	100	75	50	60	30	45
8	100	75	50	60	60	45
9	100	50	50	45	60	60
10	100	50	50	45	30	60
11	100	50	50	45	60	60
12	100	50	50	45	60	60

Table S2. The information of the top 10 sequences.

ID	Random region (40 nt)	Total reads in round 12
1	TTTGGGGATGGGCAACGGTAAAAAGGGTCAAAAGGCTTTT	89718
2	ATTTGGGTTTCGGGATAAGGGGCAAAAGGACAGGTTTTGT	56861
3	GGCGCTTGTGGTGTAAGGGTTGGGTATATGTCGTAATAGG	28436
4	GGAAAGGGGGTAGGCGGTTCAGGTTTCGGGAGGGTGGGGC	21092
5	GAATATAGGGGGTGGGGGGGTGTGTAAAGGAGGAGGGGTT	20004
6	TTTGGGGAGTTAGGGGCGAGGGAATGGGTCAGGTATTCAT	18552
7	TTGGGCAGTTAGAGGTCAGGGGTTGAAAGGTTCGGGTTTC	17050
8	CAACGGGGGGGTGGGGAGAGAAGGGTTGAGTGACAATTTG	11600
9	TTCAGGTGGGTTAGTGGTCGGGTGGTGGGGTTGTGGGGGC	10702
10	TTTGGGGTCTGGCTGGAGAGGGAAAAGGGGGATTGGCTTT	10311

Table S3. The information of the candidate aptamers.

ID	Random region (40 nt)	Free energy (kcal/mol)	Family
MBMI-01	TTTGGGGATGGGCAACGGTAAAAAGGGTCAAAAGGCTTTT	-11.47	IV
MBMI-02	ATTTGGGTTTCGGGATAAGGGGCAAAAGGACAGGTTTTGT	-12.24	III
MBMI-03	GGCGCTTGTGGTGTAAGGGTTGGGTATATGTCGTAATAGG	-10.27	I
MBMI-04	GGAAAGGGGGTAGGCGGTTCAGGTTTCGGGAGGGTGGGGC	-12.03	I
MBMI-05	GAATATAGGGGGTGGGGGGGTGTGTAAAGGAGGAGGGGTT	-9.79	I
MBMI-07	TTGGGCAGTTAGAGGTCAGGGGTTGAAAGGTTCGGGTTTC	-10.12	III
MBMI-08	CAACGGGGGGGTGGGGAGAGAAGGGTTGAGTGACAATTTG	-15.12	III

MBMI-11	CTGTGATAGAGGTAGAGTGCCCAATATCAGTGCCAATCGG	-15.18	IV
MBMI-12	GTGTCGCGGGTGGGGGTTTTATTTCGGGGATGGGGGTGGA	-10.32	IV
MBMI-15	GTTTGGGTCAGGGTGTCGGGGGAAAGGGCGGGTGCTTTTG	-10.05	I
MBMI-21	GCTCAGTCACGGTCTCGCCAATTGAACGGTATTGCCAATC	-13.28	I
MBMI-22	CAAGAAGGGGGAGGGTGGTATGTGTCGGGGGGAGGGGTGG	-12.29	I
MBMI-23	GTACAGGGAGGGGAAAGGGGTCAGTAGGTGTGCCAATTAC	-13.68	IV
MBMI-29	CAGGAAGGAGGGGGGGTTGAGGTAGGTAAAGTGTCAATAG	-12.38	III
MBMI-32	CTTTGGGTAATGGTGAGTTTCGAGAAAGGGCTGGCTTTCG	-14.16	I
MBMI-39	GTAGGGGATTTAAGGAGGGGCCTTAGGAGGGGGGGGGTAA	-13.08	IV
MBMI-51	AAGGTGGAATCGGAGGGGGTGCTTGGGTGGGGGTGGGATT	-12.21	IV
MBMI-88	GACGTCCTCCGAGGGTGGGTGGGTGGAAAGAGGCTTCGAC	-16.93	IV
MBMI-92	CTCGCGGAGGGAGGGGTTTTGGGGGGCGGGATGGTATTGG	-14.33	I
MBMI-94	AGCGGGGTTGGTTTGCGGGAGGGGGGGTGGAGGTAAGCTC	-11.35	III

Table S4. Affinity constants between the CTX-MI and candidate aptamers.

ID	Respond (nm)	K_D (μM)	K_{on} (1/ms)	K_{dis} (1/s)	X^2	R^2
MBMI-01	0.1778	0.85	6070	0.0041	0.1826	0.8424
MBMI-02	0.0662	2.21	6409	0.0141	0.0421	0.9287
MBMI-03	0.0512	3.49	10020	0.0351	0.0218	0.9631
MBMI-04	0.0360	47.7	7096	0.3382	0.2547	0.7222
MBMI-05	0.0358	39.5	43850	1.8540	0.3868	0.6697
MBMI-07	0.0246	41.5	19110	0.7937	0.7230	0.3778
MBMI-08	0.0385	12.0	18420	0.2212	0.1595	0.8303
MBMI-11	0.0455	81.0	11530	0.9331	0.5619	0.6876
MBMI-12	0.0367	32.8	10950	0.3587	0.2697	0.7474
MBMI-15	0.0259	35.2	15760	0.5540	0.3062	0.6276
MBMI-21	0.0350	30.0	15700	0.4710	0.7062	0.5764
MBMI-22	0.0481	21.2	30790	0.6514	0.2856	0.8176
MBMI-23	0.0271	35.6	33170	1.1820	0.2285	0.6994
MBMI-29	0.0285	26.5	25920	0.6854	0.3413	0.6349
MBMI-32	0.0252	25.3	35250	0.8920	0.6455	0.4380
MBMI-39	0.0467	0.43	37690	0.0162	0.0826	0.8499
MBMI-51	0.0355	14.0	22090	0.2518	0.0693	0.8982
MBMI-88	0.0276	33.3	29820	0.9944	0.3858	0.5957
MBMI-92	0.0380	0.26	31090	0.0082	0.6676	0.8648
MBMI-94	0.0320	2.33	46870	0.1090	0.0170	0.9326
MBMI-01c	0.2317	0.87	5492	0.0026	0.3409	0.8741
MBMI-02c	0.1999	1.29	5874	0.0958	0.3030	0.9206
MBMI-92c	0.1574	1.32	23772	0.0074	0.3994	0.9456

The sequences with affinity were marked in red.

Table S5. The QGRS prediction of MBMI-01c.

	Position	Length	QGRS	G-Score
A	4	23	GGGGATGGGCAACGGTAAAAAGG	19
B	4	23	GGGGATGGGCAACGGTAAAAAGG	19
C	10	26	GGGCAACGGTAAAAAGGGTCAAAAGG	19
D	10	26	GGGCAACGGTAAAAAGGGTCAAAAGG	19

The guanines that make up quadruplets are marked in red.

Table S6. The information of the mutational sequences.

ID	Sequence	Remaining GQ-core	Respond (nm)	K_D (μM)
MBMI-01c	TTTGGGGATGGGCAACGGTAAAAAGGGTCAAA AGGCTTTT	A,B,C,D	0.2331	0.729
M_1	TTTCCGGATGGGCAACGGTAAAAAGGGTCAAA AGGCTTTT	C,D	0.0834	1.52
M_2	TTTGGCCATGGCCAACGGTAAAAAGGCTCAAAA CCCTTTT	A	0.2186	0.741
M_3	TTTGGGGATGGGCAACGGTAAAAAGGGTCAAA ACCCTTTT	A,B	0.1901	1.56

The mutated nucleotides are marked in red.

Table S7. The QGRS prediction of the mutational sequences.

		Position	Length	QGRS	G-Score
M_1	C	10	26	GGGCAACGGTAAAAAGGGTCAAAAGG	19
	D	10	26	GGGCAACGGTAAAAAGGGTCAAAAGG	19
M_2	A	4	23	GGGGATGGGCAACGGTAAAAAGG	19
M_3	A	4	23	GGGGATGGGCAACGGTAAAAAGG	19
	B	4	23	GGGGATGGGCAACGGTAAAAAGG	19
	E	5	22	GGGGATGGGCAACGGTAAAAAGG	19

The guanines that make up quadruplets are marked in red.

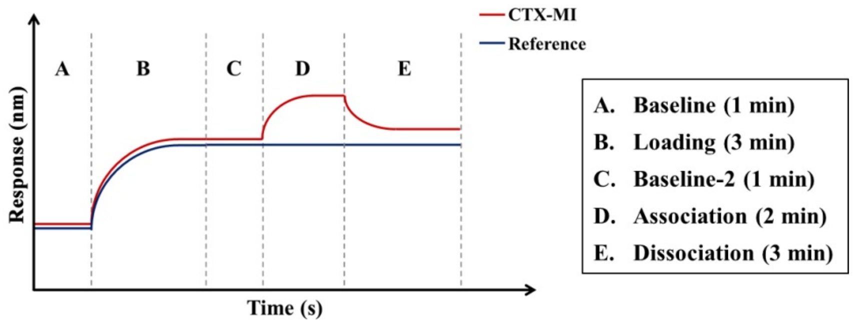

Figure S1. The procedure of the BLI assay.

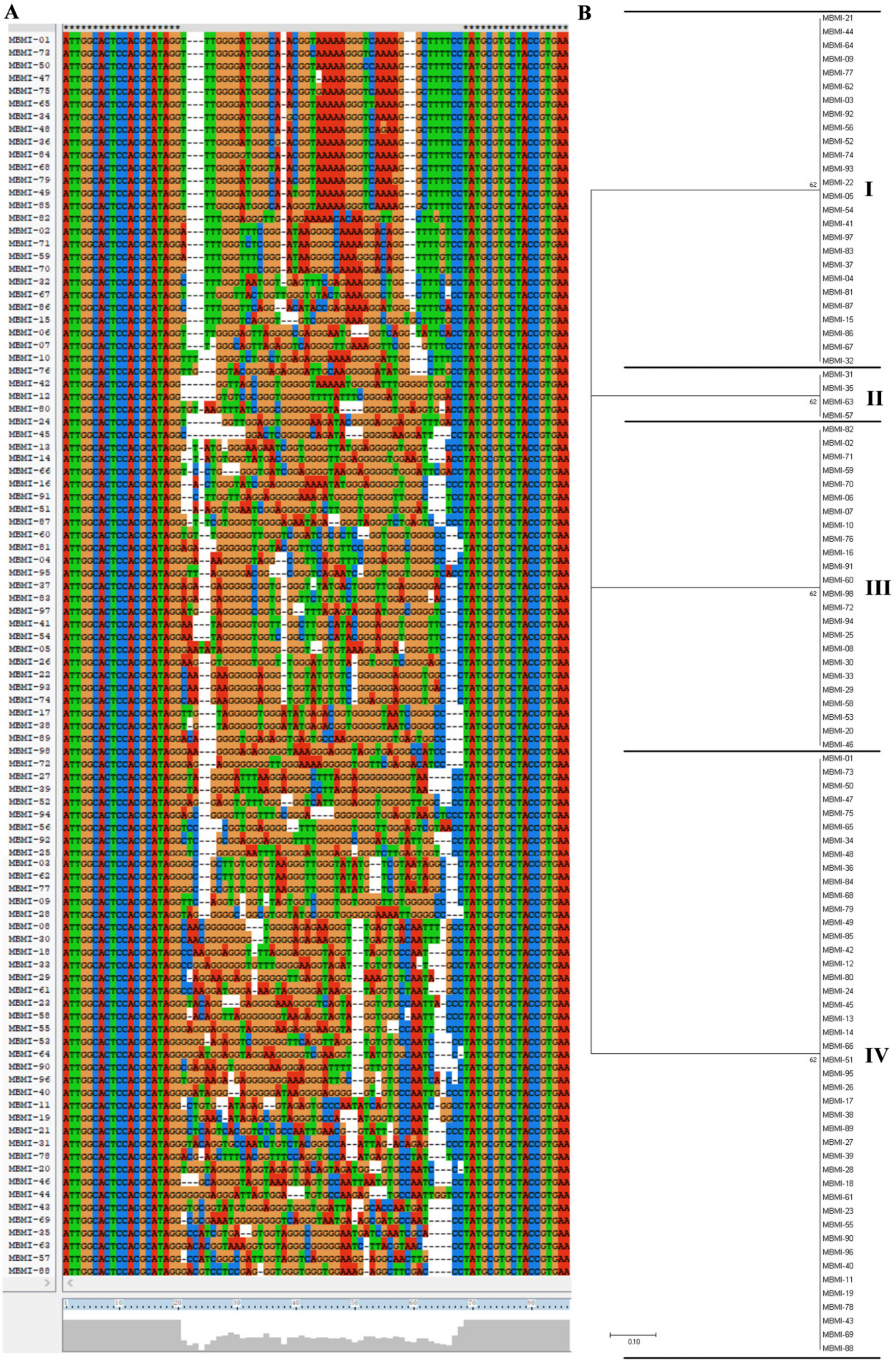

Figure S2. The information of the alternative aptamers. (A) Multiple sequence alignment by Clustal X 2.1. The full-length of the sequences (80 nt) were used here. (B) The N-J tree constructing by MEGA 7. These sequences were grouped into 4 families (I-IV).

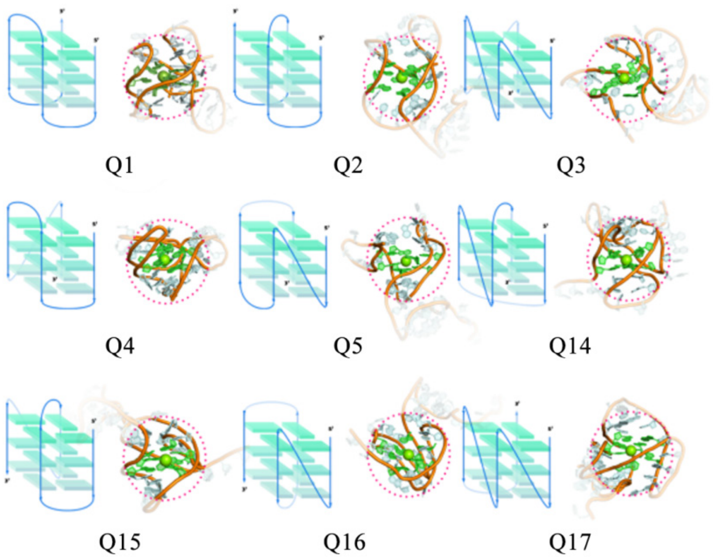

Figure S3. The candidate full-length models of each possible GQ-core.

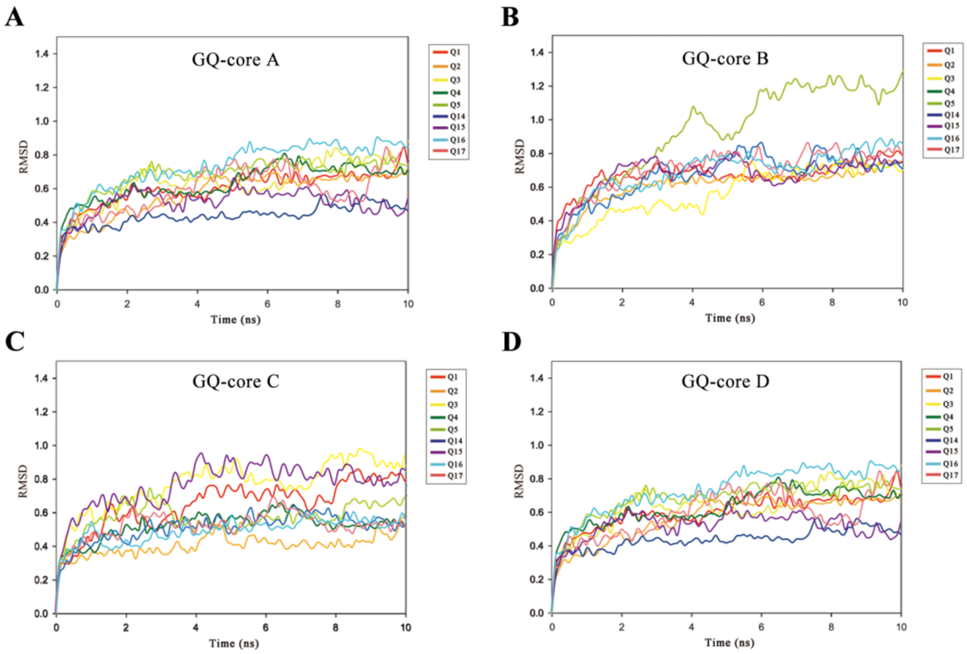

Figure S4. RMSD vs simulation time in the constant-temperature phase.

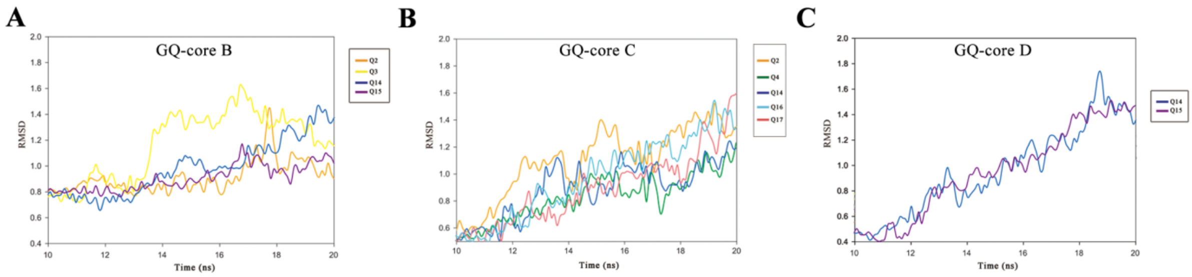

Figure S5. RMSD vs simulation time in the heating phase.

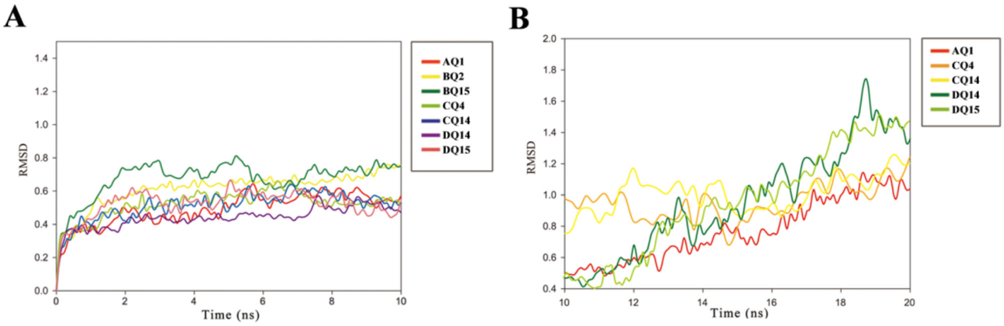

Figure S6. RMSD vs simulation in the second round of TdMD simulation. (A) The results in the constant-temperature phase. (B) The results in the heating phase.

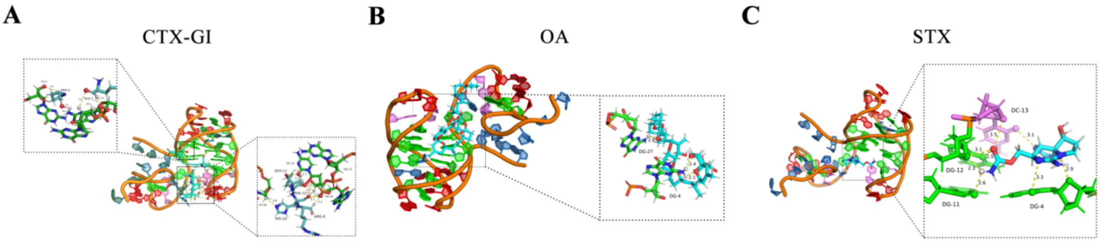

Figure S7. Three-dimensional view of the interaction between MBMI-01c and the ligands (A. CTX-GI; B. OA, C. STX). The hydrogen bond between the docking site of MBMI-01c and the ligand is depicted by a yellow dashed line, and the distance was marked next to the dashed line.
